# Cross-platform opinion dynamics in competitive travel advertising: A coupled networks’ insight

**DOI:** 10.3389/fpsyg.2022.1003242

**Published:** 2022-10-28

**Authors:** Jia Chen, Haomin Wang, Xiangrui Chao

**Affiliations:** ^1^School of Business Administration, Faculty of Business Administration, Southwestern University of Finance and Economics of China, Chengdu, China; ^2^School of Management Sciences, Southwestern University of Finance and Economics of China, Chengdu, China; ^3^Business School, Sichuan University, Chengdu, China

**Keywords:** coupled network propagation, competitive travel advertising, opinion leaders, opinion guidance, simulation model

## Abstract

Social media platforms have become an important tool for travel advertisement. This study constructs the bounded confidence model to build an improved cross-platform competitive travel advertising information dissemination model based on open and closed social media platforms. Moreover, this study examines the evolution process of group opinions in cross-platform information dissemination with simulation experiments. Results reveal that based on strong relationships, the closed social media platform opinion leaders better guide in competitive travel advertising and can bring more potential consumers to follow. However, being an opinion leader on an open social media platform will not result in more consumer following.

## Introduction

With the growing popularity of social networks, many tourists like to share their travel experiences (Lin et al., 2017; Liu et al., 2018). These social media platforms, such as TripAdvisor, Weibo, WeChat, and Ctrip, promote trust fusion among users through sharing, recommendation, communication, and other elements (Kah and Lee, 2014; Pötzschke and Braun, 2017; De Vries et al., 2017; Göbel and Munzert, 2018). This weakens the purpose of business information, makes social media publicity more convincing, and improves the company’s marketing efficiency (Hajli and Sims, 2015). For example, online travel reviews on social platforms, including reviews of hotels and restaurants, have become an essential source of information for consumers making travel plans, thus enhancing hotel and restaurant advertising communication effects (Zhang et al., 2016). Compared with traditional information dissemination channels, the number of users of social platforms is growing, and it has become the preferred method for users to receive travel information. Indeed, public opinion has emerged as a critical norm in the dissemination of social information (Zhang et al., 2018; Kim et al., 2019; Shmargad and Sanchez, 2020; Ju et al., 2022).

As the primary means of disseminating public opinion, social media platforms are an important channel for Internet users to obtain much information. Therefore, as the second-largest online advertising platform, social media has attracted about 40% of advertisers to increase their advertising budgets, formulate personalized advertising content, share and spread, and generate various advertising effects (Nasir et al., 2021). However, due to the complexity of the Internet and social networks, product information dissemination is not always ideal and productive. Users cannot accurately judge the authenticity and accuracy of information; hence, product information is not widely disseminated, thereby influencing the advertising dissemination (Zhang et al., 2018). Prior research discovered that in the process of disseminating advertising information on social media platforms, opinion leaders are those who can influence other consumers’ attitudes toward products by sharing their experiences with products or services and prompting them to make purchases, such as the big V travel blogger on Weibo (Eck et al., 2011). Moreover, prior study has found that opinion leaders typically have reliable knowledge in a specific field, a certain social and economic position, and the ability to attract others (Lazarsfeld et al., 1968). In addition, opinion leaders have high exposure in information dissemination and can influence other social users (Rogers, 2003). Therefore, opinion leaders have a significant impact on consumers’ purchasing decisions on social media platforms. Research shows that 49% of users will rely on product recommendations from opinion leaders, and 40% will eventually purchase products recommended by opinion leaders (Karp, 2016). Therefore, using opinion leaders on social media platforms to promote products and guide consumers’ opinions has become an important market strategy for tourism businesses.

Moreover, previous studies have examined the impact of opinion leaders on users’ opinions on a single social platform (Eck et al., 2011; Dong et al., 2017; Zhao et al., 2018). However, the connection in the real world is becoming increasingly complex, and various information, such as hotel and restaurant reviews, are already flowing in multiple networks. These networks no longer exist in isolation, but they are interdependent and linked by structural and dynamic characteristics. These coupled systems can be found exist in multiple social platforms. For example, users use WeChat, Ctrip, and other apps to exchange information and Weibo, TripAdvisor, and other apps to share information and communicate with others.

Obviously, online travelers can be active in multiple social networks, and they can access and exchange information through multiple physical and social networks that intersect. Therefore, information is now distributed across multiple coupled networks rather than a single-platform network. The information dissemination of the coupled network has become more complex as the functions of social platforms have been upgraded and the range of travel users has expanded. As an important node of information dissemination, travel users directly determine the impact of information dissemination in the network. Travel users in a coupled network will receive information from multiple social network platforms, thus broadening the scope of information dissemination. If information dissemination in the coupled network is not managed, it may result in an accelerated attenuation of information propagation, thus reducing the effect of travel marketing information propagation (Zhang et al., 2018). Therefore, according to the dissemination law of coupled network information, establishing a model based on actual characteristics and constructing a coupled dissemination system are important ways to explore multi-platform travel information dissemination.

To investigate the influence of coupled network opinion leaders on consumers, this study builds a propagation model of competitive travel advertising information in coupled networks and analyzes the guiding process of opinion leaders in coupled networks to consumers’ opinions based on the Hegselmann–Krause (HK) model of dissemination. Therefore, this study contributes in the following ways. First, we more realistically simulate the coupling propagation process of travel information by analyzing the dissemination and network characteristics of the two networks. Second, we analyze the dissemination mechanism of travel competitive advertising information in coupled networks and make recommendations for information dissemination on multi-platform networks in this manner.

The rest of this paper is structured as follows. Section 2 provides theoretical context and opinion dynamics. Section 3 develops an integrated public opinion dynamics model to examine the opinion evolution law of the coupled network’s opinion leaders and followers. Section 4 shows the dissemination effect of competitive travel advertising in the coupling network under opinion leaders with the computer simulation. Finally, Section 5 presents the conclusion and discussions.

## Literature review

In this section, we present definitions and properties of opinion leaders, and analysis the influence of opinion leaders. Then we analyze the characteristics of information dissemination, in addition, we focus on the opinion model in information dissemination, summarize the dissemination models in a single platform and multiple platforms, and analyze the necessity of research on multi-layer network cross-layer dissemination.

### The influence of opinion leaders

Previous studies have shown that opinion leaders have an important influence on social platforms (Chen et al., 2016, 2021; Zhao et al., 2018). Opinion leaders, who are also social media influencers, usually utilize their ability to be “trusted person” in social media to influence brand awareness and the purchase decisions of large consumers (Cheng et al., 2019; Hudders et al., 2020). Furthermore, opinion leaders are social media micro-celebrities with a large following and significant influence on their audiences. This position on social media enables them to communicate the brand’s marketing message and influence consumer opinions (Delbaere et al., 2020). In the dissemination of electronic word-of-mouth (eWOM), opinion leaders often have definite, unwavering target opinions; their purpose is to influence other followers’ opinions, and they are not affected by followers in the opinion update process (Zhao et al., 2018). In the opinion dynamics, opinion leaders, with more power, expertise, and positions, can affect other agents’ opinions and achieve consensus or polarization of group decision-making (Dong et al., 2017; Zhang et al., 2020). Because opinion leaders’ extensive exposure to mass media and close ties to change agents, which makes He/she becomes an influential social participant (Rogers, 2003). Therefore, the flow of public opinion and information is transferred from the mass media to the general public through the mediating role of opinion leaders (Lazarsfeld et al., 1944; Katz, 1957). From the perspective of opinion dynamics theory, according to the network structure, opinion leaders can play a key role in the network, most likely to influence the information flow of a large number of followers (Das et al., 2014). Simultaneously, several opinion dynamic models have been established and various experiments have been conducted to investigate the role of opinion leaders in the evolution of public opinion (Zhao and Kou, 2014; Chen et al., 2016, 2021). Opinion leaders will gradually shift public opinion to the desired target through micro-interaction during the opinion evolution process. Especially when they have similar opinions, gradually and intentionally changing others’ opinions in the desired directions becomes easier (Afshar and Asadpour, 2010; Fan and Pedrycz, 2016).

### Information dissemination

Travel information, such as online travel reviews, is a crucial source of information for tourists and facilitate their travel decisions (Duverger, 2013). The dissemination of travel information is essentially disseminating public opinion. Much research progress has been made on public opinion dissemination in the social networks. The spread of public opinion and infectious diseases are similar; thus, many scholars used the infectious disease model to study the public opinion dissemination. For instance, Wang et al. (2019) built a discrete communication model to discuss the spread of public opinion using the infectious disease susceptible, infected, and recovered model. They combined the hedging effects of negative and positive information. Their findings show that, in disseminating public opinion, netizens, the media, and the government will continuously optimize their strategies based on their own interests and information feedback. Opinion dynamics models, when applied to information dissemination, primarily examine how individuals interact and update their opinions in social networks. The opinion dynamics model is mainly used to describe specific aspects of the social behavior of a number of individuals and to simulate how the opinions of a group of groups evolve over time (Castro et al., 2018). There have been various approaches to analyze the process of changing these opinions, based on given various assumptions in the process. Using the continuous opinion and discrete actions model, Martins (2008) examined the discrete behavior of individual opinion interactions and deeply explored the impact of interaction rules on opinion evolution. Meanwhile, other researchers have examined the evolution of public opinion using a variety of public opinion dynamics models, for example, the Voter model, the DeGroot model, and the HK model, the details are shown in Table 1. As can be seen from Table 1, the opinion dynamics of a single social network has been extensively studied, both in terms of formation and evolution and opinion consensus reaching process, and these studies provide in-depth insights into the evolution of descriptions’ opinions in an isolated network. In fact, public opinion dissemination is an overly complex dynamic process, and describing the dissemination process clearly is difficult. Especially with the development of information technology, information is no longer disseminated on a single platform but cross-disseminated in multiple platforms, making the description of public opinion dissemination increasingly complicated. However, there are few studies on the dissemination of cross-disseminated in multiple platforms. For example, the cross-layer propagation of single information online and offline is analyzed through the HK model (Ding et al., 2017; Dong et al., 2021; Ju et al., 2022). Because the cross-layer communication of online platforms is faster and more common. Therefore, based on the HK model, this paper constructs a cross-layer coupling propagation model of tourism advertising on open social media platforms and closed social media platforms, and further analyzes the information propagation in multi-layer networks.

**Table 1:** Opinion dynamics models at different social networks.

Application fields	Research object	Basic models	References
Single social network	Opinion formation and evolution	Bounded Confidence Model and Extensions	Zhao and Kou (2014), Zhao et al. (2018), Li et al. (2020), Hou et al. (2021), Li et al. (2021), Zhan et al. (2022)
		Voter model	Klamser et al. (2017), Jiao and Li (2021)
		Degroot model	Jia et al. (2017), Castro et al. (2018), Zhou et al. (2020), Wu et al. (2022)
	Opinion consensus reaching process	Bounded Confidence Model and Extensions	Pérez et al. (2018), Zhang et al. (2019), Zhang et al. (2020), Zhang et al. (2021)
		DeGroot model	Ding et al. (2019), Li and Wei (2019), Liang et al. (2022)
		Voter model	Gastner et al. (2018), Herrerías-Azcué and Galla (2019)
Multiple social networks	Opinion formation and evolution	Bounded Confidence Model and Extensions (online and offline)	Ding et al. (2017), Dong et al. (2021), and Ju et al. (2022)
		Voter model	Diakonova et al. (2016)
		SIR model	Zhang et al. (2018) and Jankowski and Chmiel (2022)
		Cross-network propagation model	Li and Wang (2022)

## Construction of a coupled two-layer online social network

The purpose of this paper is to analyze the dissemination of tourism advertising information in a multi-layer coupled network. Studies have shown that social network structure has an important impact on the dissemination of information (Zhang et al., 2018; He et al., 2021). Therefore, this section analyzes the structure of online social media platforms, and builds a two-layer social network.

### Analysis of online social network structure

The dissemination of information is closely related to the structure of social networks. Complex network theories and methods are widely used in dissemination dynamics (Li et al., 2015; He et al., 2021). Existing research has shown that network topologies, such as WeChat and Facebook, have a critical impact on public opinion dissemination (Zhang et al., 2018; Mandal et al., 2020; Li and Wang, 2022).

There are numerous social media platforms available today, and their structures vary greatly. For example, WeChat, Weibo, Facebook, Twitter, Ctrip, and other platforms have vastly different user connections and usage frequency. Some social media platforms, such as Weibo and Ctrip, only require one-way contact between users to communicate, even if they are unfamiliar with each other. These social platforms’ network structure can be considered to have weak relationship strength. Meanwhile, in some social media platforms, such as WeChat and Facebook, users can only make contact through mutual authentication, which means they can only add friends and exchange information through authentication. Users on such social media platforms have stronger relationships and a higher level of trust. Previous research has classified existing social media platforms into two major groups (Li and Wang, 2022).

#### Open social medias

The connection between users is built based on one-way authentication. The users here usually have various friends, shallow social relationships, and weak friendship. In open social media, users can freely establish interactive relationships, and the number of users connected is large. Like Weibo, open social media users can forward other’ Weibo content to their own Weibo through the forwarding function and like and reply to the content. Moreover, users usually have strong flexibility, which can be a one-way or two-way relationship. A weak relationship network of “radiation” is formed through the user’s attention, which triggers the “secondary radiation propagation” of information. The structure of open social media is the directed, scale-free networks (Barabasi and Albert, 1999). Therefore, this paper chooses BA-directed scale-free networks to simulate these social media platforms.

#### Closed social medias

The establishment of the connection relationship between users is achieved through mutual authentication. Usually, the number of users’ friends is small, and the mutual trust is higher. The addition of user friends on a closed social media platform is mostly recommended by other users or searched for by the system. Thus, the growth of this network structure is random. Users add friends through mobile phone numbers, QQ friends, and so on, similar to WeChat, thus forming a peer-to-peer communication mode. This is a social model based on offline acquaintances who have a strong bond with one another. The majority of group communication occurs through the formation of WeChat groups, and the information in the user’s circle of friends can be seen by other friends, resulting in information group communication. These communication methods rely on a “circle” network with strong relationships. Additionally, information dissemination has privacy, and individuals have a higher degree of trust. The structure of these social media is a typical undirected BA scale-free network (Traud et al., 2012). Additionally, this paper chooses an undirected BA scale-free network to simulate these social media platforms.

### Construction of two-layer coupling online social networks

**Figure 1:**
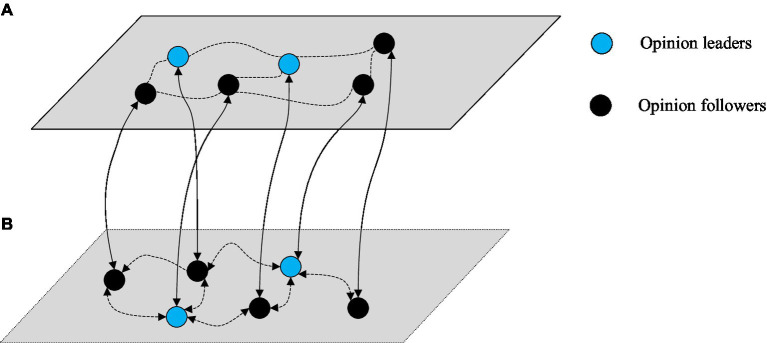
**(A,B)** Two-layer coupled network structure.

Social diversity has become a defining feature with the new media development. This means that public opinion spreads not only on one social media platform but also across networks in multiple platforms. For example, users may capture Ctrip travel information and forward it to WeChat; similarly, WeChat travel experiences may be forwarded to Twitter. To explore cross-platform information dissemination, we define two online social networks, namely, network A and network B. Network A is a closed social media platform, whereas network B is an open social media platform. In Figure 1, the edges between nodes in each layer represent social relationships in the platform. The connecting edge between networks A and B indicates that a user can have accounts in multiple social networks. Furthermore, in this coupled network, the correspondence between networks A and B is one-to-one; others are ignored (Li and Wang, 2022). Nodes in networks A and B are connected at random. In addition, the opinion leader in network A may be a follower in network B. Similarly, an opinion leader in network B could be an opinion follower in network A.

In this coupled network, the following are some assumptions:

1. Each user has one and only one account in networks A and B. This means that individuals on open social media platforms and closed social media platforms can receive any information from both platforms at the same time.
2. The user’s addition or deletion is not considered; that is, the network is static.
3. The states of the same node in two different network layers are allowed to be different.
4. A group of opinion leaders exists in networks A and B, and their status is affected only by the opinions of the target travel advertisement;
5. Once the opinion leader group publishes an opinion, networks A and B users can receive the message immediately.

## Design of competitive advertising propagation model in coupled network

In social media platforms, potential consumers will always trust individuals with similar opinions (Liu et al., 2015). Therefore, the HK model can better describe the dynamic evolution process of consumer opinions. The original HK model is defined as:

Let $X\left(t\right)=\left\{x_{1}\left(t\right),x_{2}\left(t\right),\ldots x_{N}\left(t\right)\right\}$ be the set of opinions of individual *i* at time *t*. For the case $|x_{i}\left(t\right)-x_{j}\left(t\right)|\leq \varepsilon$ , the opinion update rule of individual *i* at time *t* + 1 is as follows (Heselmann and Krause, 2002):


(1) $$x_{i}\left(t+1\right)=\frac{\sum_{j:|x_{i}\left(t\right)-x_{j}\left(t\right)|\leq \varepsilon }\sigma_{ij}x_{j}\left(t\right)}{\sum_{j:|x_{i}\left(t\right)-x_{j}\left(t\right)|\leq \varepsilon }\sigma_{ij}}$$

where, *ε* is the bounded trust level of the individual, and *σ_ij_* is the weight that individual *i* assigns to the individual *j* at time.

Under the framework of bounded trust theory, we construct two competitive opinion groups in a two-layer coupled network to analyze the evolution process of individuals. Without loss of generality, this paper assumes an opinion leader group exist in both networks A and B. Each opinion leader group represents opposing advertising opinions for competitive products. Therefore, the target advertising opinion of the opinion leader group in network A is 1, whereas the target travel advertising opinion of the opinion leader group in network B is −1.

The dissemination of advertising opinions depends on the network’s topology (Boccaletti et al., 2006). Assuming that [*a_ij_*]_*N*×*N*_ is the adjacency matrix of network A, we determine that *a_ij_* = 1 means a connection exists between individuals *i* and *j* in network A; otherwise, *a_ij_* = 0 denotes no connection between individuals *i* and *j*. Meanwhile, [*b_ij_*]_*N*×*N*_ is the adjacency matrix of network B: if *b_ij_* = 1, a connection exists between individuals *i* and *j* in network B; otherwise, *b_ij_* = 0 denotes no connection. If individuals *i* and *j* are connected, individual *i* can receive an opinion from individual.

According to the HK model, if $|x_{i}\left(t\right)-x_{j}\left(t\right)|\leq \varepsilon$ , the rule of opinion leader groups in network A is defined as:


(2) $$\begin{array}{l} x_{i}^{L_{1}}\left(t+1\right)=\\ \left(1-w_{1}\right)\left[\frac{p_{i}}{N_{i}^{L_{1}}\left(t\right)}\overset{N_{1}+N_{2}}{\underset{j=N_{1}+1}{\sum }}\delta_{ij}^{1}\left(t\right)a_{ij}x_{j}\left(t\right)+\left(1-p_{i}\right)x_{i}\left(t\right)\right]+w_{1}d_{1} \end{array}$$

where, *i* = *N*_1_ *+* 1*,…,N*_1_ *+ N*_2_, $\delta_{ij}^{1}\left(t\right)=\left\{\begin{array}{l} 0,\mathrm{otherwise}\\ 1,\;||x_{j}\left(t\right)-x_{i}\left(t\right)||\;\leq \varepsilon_{1} \end{array}\right.$ , *ε*_1_ is the bounded confidence level of the individuals in the network A. $\frac{1}{N_{i}^{L_{1}}\left(t\right)}=\overset{N_{1}+N_{2}}{\underset{j=N_{1}+1}{\sum }}\delta_{ij}^{1}\left(t\right)a_{ij}$ represents the number of neighbors of opinion leaders in network A. As aforementioned, *a*_*ij*_ represents the connection of network A, which is 0 or 1. *w*_1_ is the influence weight of the target advertisement in the network A. *p_i_* represents the level to which the individual is affected by the opinion from the network A. Therefore, 1 − *p_i_* is the individual’s self-confidence degree when receiving the opinion, and *d*_1_ is the target travel advertisement opinion value in the network A. This shows that the opinions of opinion leaders are mainly influenced by targeted advertisements and other opinion leaders in the same layer group.

As aforementioned, the rule of opinion leader groups in network B is defined as follows:


(3) $$\begin{array}{l} x_{i}^{L_{2}}\left(t+1\right)=\\ \left(1-w_{2}\right)\left[\frac{q_{i}}{N_{i}^{L_{2}}\left(t\right)}\overset{N}{\underset{j=N_{1}+N_{2}+1}{\sum }}\delta_{ij}^{2}\left(t\right)b_{ij}x_{j}\left(t\right)+\left(1-q_{i}\right)x_{i}\left(t\right)\right]+w_{2}d_{2} \end{array}$$

where, *i* = *N*_1_ *+ N*_2_ *+* 1*,…,N*, $\delta_{ij}^{2}\left(t\right)=\left\{\begin{array}{l} 0,\mathrm{otherwise}\\ 1,\;||x_{j}\left(t\right)-x_{i}\left(t\right)||\leq \varepsilon_{2} \end{array}\right.$ , *ε*_2_ is the bounded confidence level of the individuals in the network B. $\frac{1}{N_{i}^{L_{2}}\left(t\right)}=\overset{N}{\underset{j=N_{1}+N_{2}+1}{\sum }}\delta_{ij}^{2}\left(t\right)b_{ij}$ represents the number of neighbors of opinion leaders in network B. *b_ij_* represents the connection of network B, which is 0 or 1. *w*_2_ is the influence weight of the target advertisement in network B. *q_i_* represents the level to which the individual is affected by the opinion from network B. Therefore, 1 − *q_i_* is the individual’s self-confidence degree when receiving the opinion, and *d*_2_ is the target travel advertisement opinion value in the network B.

The opinion update model for opinion followers in networks A and B is:


$$\begin{array}{c} x_{i}^{F}\left(t+1\right)=p_{i}[\alpha \frac{1}{N_{i}^{L_{1}}\left(t\right)}\overset{N_{1}+N_{2}}{\underset{j=N_{1}+1}{\sum }}\delta_{ij}^{1}\left(t\right)a_{ij}x_{j}\left(t\right)\\ +\left(1-\alpha \right)\frac{1}{N_{i}^{F_{1}}\left(t\right)}\underset{j\in F_{1}}{\sum }\delta_{ij}^{1}\left(t\right)a_{ij}\left(\begin{array}{l} q_{i}\frac{1}{{N^{\prime }}_{j}^{F_{2}}\left(t\right)}\underset{k\in F_{2}}{\sum }\delta_{jk}^{\prime }\left(t\right)b_{jk}x_{k}\left(t\right)\\ +\left(1-q_{i}\right)x_{j}\left(t\right) \end{array}\right)]\\ +q_{i}[\alpha \frac{1}{N_{i}^{L_{2}}\left(t\right)}\overset{N}{\underset{j=N_{1}+N_{2}+1}{\sum }}\delta_{ij}^{2}\left(t\right)b_{ij}x_{j}\left(t\right)\\ +\left(1-\alpha \right)\frac{1}{N_{i}^{F_{2}}\left(t\right)}\underset{j\in F_{2}}{\sum }\delta_{ij}^{2}\left(t\right)b_{ij}\left(\begin{array}{l} p_{i}\frac{1}{{N^{\prime \prime }}_{j}^{F_{1}}\left(t\right)}\underset{k\in F_{1}}{\sum }\delta_{jk}^{\prime \prime }\left(t\right)a_{jk}x_{k}\left(t\right)\\ +\left(1-p_{i}\right)x_{j}\left(t\right) \end{array}\right)]\\ +\left(1-p_{i}-q_{i}\right)x_{i}^{F}\left(t\right) \end{array}\left(4\right)$$

where, $\delta_{jk}^{''}\left(t\right)=\left\{\begin{array}{l} 0,\mathrm{otherwise}\\ 1,\;||x_{j}\left(t\right)-x_{k}\left(t\right)||\leq \theta_{1} \end{array}\right.$ , $\delta_{jk}^{'}\left(t\right)=\left\{\begin{array}{l} 0,\mathrm{otherwise}\\ 1,\;||x_{j}\left(t\right)-x_{k}\left(t\right)||\leq \theta_{2} \end{array}\right.$ , *θ*_1_ and *θ*_2_ represents the inter-layer propagation thresholds of networks A and B. That is, the threshold for network A users to spread their opinion to network B after accepting their opinion is *θ*_1_, whereas the threshold for network B users to spread their opinion to network A after accepting their opinion is *θ*_2_. Target advertising opinions spread cross-network in coupled networks A and B when the values of *θ*_1_ and *θ*_2_ are less than or equal to the bounded confidence level, respectively. *α* is the degree of followers affected by the group of opinion leaders in networks A and B. *F*_1_ and *F*_2_ represent the set of followers of the opinion leaders’ opinion dissemination across layers in networks A and B, where $F_{1}=\left\{1,\ldots ,N_{1}\right\}\cup \left\{N_{1}+N_{2}+1,\ldots ,N\right\}$ , $F_{2}=\left\{1,\ldots ,N_{1}+N_{2}\right\}$ , and $N_{i}^{F_{1}}\left(t\right)=\underset{j\in F_{1}}{\sum }\delta_{ij}^{1}\left(t\right)a_{ij}$ , $N_{i}^{F_{2}}\left(t\right)=\underset{j\in F_{2}}{\sum }\delta_{ij}^{2}\left(t\right)b_{ij}$ , $N_{j}^{'F_{2}}\left(t\right)=\underset{k\in F_{2}}{\sum }\delta_{jk}^{'}\left(t\right)b_{jk}$ , and $N_{j}^{''F_{1}}\left(t\right)=\underset{k\in F_{1}}{\sum }\delta_{jk}^{''}\left(t\right)a_{jk}$ .

## Simulation analysis of competitive advertising in coupling networks

The opinion dynamics model usually describes the evolution of group opinions with a simulation (Castro et al., 2018), therefore, this study uses a computer simulation method to analyze the group opinions dynamic evolution process of opinion leaders who promote competitive travel advertisements in coupled network. Some initial assumptions are applied in the experiments:

1. The coupled network has 1,000 nodes: networks A and B have 10 opinion leaders, respectively, and the remaining nodes are followers.
2. The initial opinions of opinion leaders and followers all obey the uniform distribution on [−1,1].
3. The confidence levels of individuals in networks A and B are *ε*_1_ = *ε*_2_ = 0.5; the level of individuals affected by the opinions of others in networks A and B is *q_i_* = *q_i_* = 0.5; followers in networks A and B are influenced by opinion leaders *α* = 0.5.
4. The threshold for network A (B) users to spread the opinion to network B (A) after accepting opinion is *θ*_1_ = *θ*_2_ = 0.3.
5. The opinion values of target travel advertisements in networks A and B are *d*_1_ = 1, *d*_2_ = −1; the weight of target travel advertisements is *w*_1_ = *w*_2_ = 0.5.

**Figure 2:**
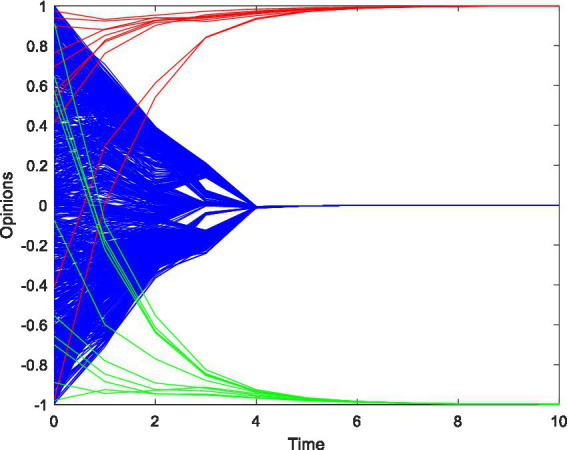
The opinion evolution with initial conditions.

As shown in Figure 2, the red, green, and blue lines represent opinion leader group in network A, opinion leader group in network B, and opinion followers, respectively. When the same travel advertising weight influences the two network platforms, number of opinion leaders, and confidence level, the opinion leaders in the two networks quickly converge to the target travel advertising opinions, and the followers’ opinions quickly converge to the middle opinion value of 0. This shows that under the influence of factors, such as the same travel advertising intensity, potential followers will not appear to be biased toward a certain network of travel advertising opinions.

However, in a closed social network, individuals usually have a strong relationship with each other than in open social network, so they usually have a higher level of trust with each other (Li and Wang, 2022) and a higher level of confidence in others. Therefore, assume that *ε*_1_ = 0.7, *ε*_2_ = 0.5 and other parameters are as above.

**Figure 3:**
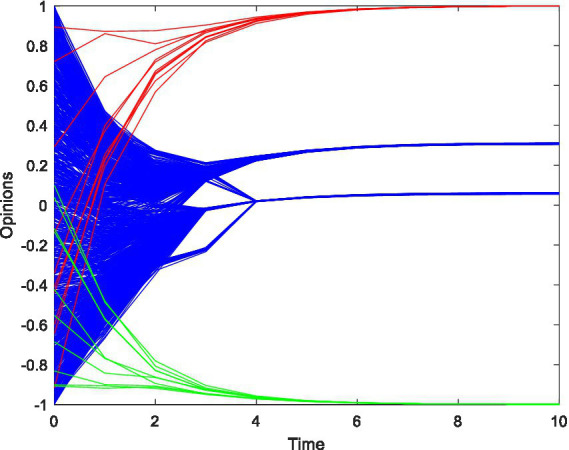
Opinion evolution with changes in confidence level.

Figure 3 shows that, in a coupled network, followers’ opinions eventually converge in the intervals [0.2, 0.4], and [0, 0.2], indicating that followers’ opinions generally tend to target travel advertising of closed social media while completely ignoring open social media advertising. This demonstrates that, when all other conditions remain constant, travel advertisements in closed social media are more likely to be accepted by potential consumers than open social media, thus bringing more potential consumers to follow in both social media platforms.

Closed social media travel advertisements can bring more potential consumers to follow. How then can open social media companies take measures to further enhance potential consumers’ recognition of their advertisements? The first measure is to increase the open social media travel advertising weight.

**Figure 4:**
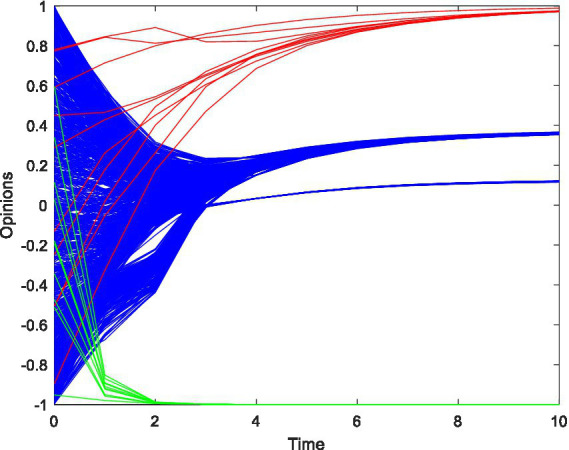
The opinion evolution with changes of travel advertisement weight.

In Figure 4, the confidence level is *ε*_1_ = 0.7, *ε*_2_ = 0.5, the weight of advertisement is *w*_1_ = 0.3, *w*_2_ = 0.9, and other parameters are as aforementioned. The results reveal that with the increase in travel advertisement weight, the opinions of opinion leaders in network B quickly converge to the target travel advertisement opinion value of −1. However, followers’ opinions eventually converge to an interval greater than 0. This demonstrates that in cross-platform communication, opening social media by increasing the weight of travel advertising will not result in followers recognizing the target travel advertisements. They continue to rely on closed social network travel advertising information. Therefore, increasing the weight of travel advertising is ineffective.

**Figure 5:**
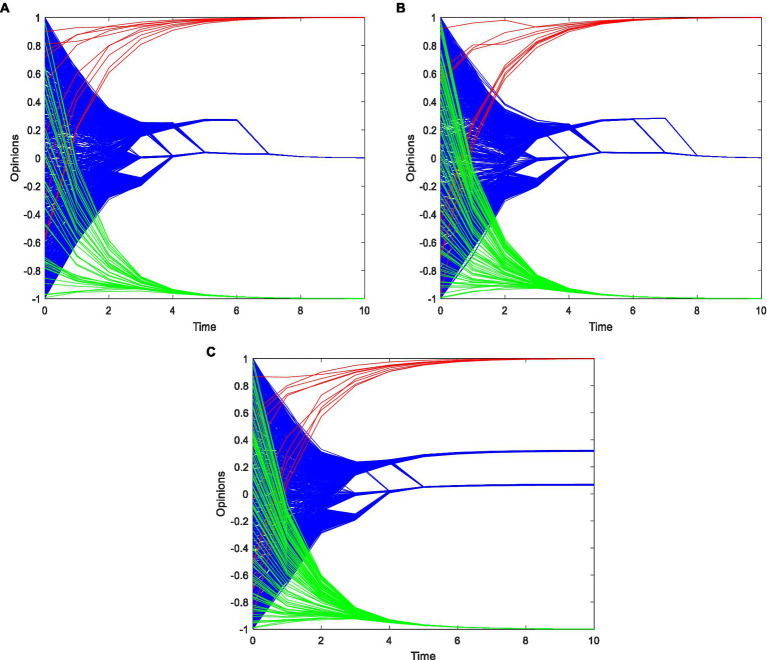
The opinion evolution with opinion leader. **(A)** *N*_1_ = 10, *N*_2_ = 40. **(B)** *N*_1_ = 10, *N*_2_ = 80. **(C)** *N*_1_ = 10, *N*_2_ = 120.

To analyze the impact of the number of opinion leaders on potential users in the coupled network, this study increases the number of opinion leaders on open social platforms. The results are shown in Figure 5. The confidence level is *ε*_1_ = 0.7, *ε*_2_ = 0.5, and the weight of the advertisement is *w*_1_ = 0.5, *w*_2_ = 0.5. The number of opinion leaders is *N*_1_ = 10*, N*_2_ = 40, *N*_2_ = 80, *N*_2_ = 120, respectively. Results reveal that as the number of opinion leaders on the open platform grows, followers’ opinions gradually converge to the middle opinion value of 0, and they no longer only follow to the target travel advertisement of closed social platforms. However, as the number of opinion leaders grows, the opinion value of followers returns to the interval above 0.

This result demonstrates that a moderate increase in the number of opinion leaders on open platforms can appropriately guide the evolution of consumers’ opinions on travel advertising in the coupled network. However, it cannot finally make consumers recognize the travel advertising of open social platforms.

## Conclusion and discussion

This paper investigates the process by which potential consumers’ opinions evolve in a coupled network under the influence of competitive travel advertising promoted by opinion leaders. The evolution of consumers’ opinions in real closed social media and open social media is simulated in a computer by building a bounded confidence opinion dynamics model of individuals in a cross-platform coupled network. This study provides a scientific strategy for travel advertising or WOM promotion on multiple social media platforms.

The results can be summarized as follows:

1. For multiple social media platforms, closed social media have better cross-platform guidance effects in the process of cross-platform dissemination of competitive travel advertisements, whereas open social media are less effective than closed social media. The findings of this study differ from the findings of the single-platform opinion evolution study (Zhao et al., 2018). In a single platform, the final followers’ opinion is symmetrically distributed with opinion interval, and no apparent bias exists toward any one opinion leader subgroup. The results show that increasing the confidence level did not significantly improve the opinion leaders’ influence. However, this study found that the final opinion of followers clearly favors opinion leaders in closed social media platforms in a coupled network. This demonstrates that in a coupled network, the followers’ choice of leaders is influenced by their confidence level.
2. Because competitive travel advertisements are spread across the coupled platforms, increasing the weight of travel advertising will not improve the cross-platform guidance effect of advertising. This finding differs from that of previous studies (Previte, 1999; Coker, 2017). Prior research has found that competing advertisement influence should be in an effective range; otherwise, the advertisement will suffer a negative effect (Previte, 1999; Coker, 2017). However, in the coupled network, increasing the influence weight of travel advertising will not result in more cross-platform consumers following. The possible reason is that in cross-platform information dissemination, consumers are more cautious about advertisements and more prone to question the advertisement information (Priester and Petty, 1995; Yang and Hsu, 2017). Although the influence weight of travel advertisements has increased, influencing consumers’ decision-making is not enough.
3. Opinion leaders of open social media play a lesser role in cross-platform information dissemination than closed social network platforms. Previous studies have shown that opinion leaders on open social platforms have a critical influence on the scale of information dissemination on a single platform (Luqiua et al., 2019; Wang et al., 2020). However, this paper found that, in the coupled network, although the open platform opinion leaders will affect the potential consumers’ opinions, it ultimately failed to make potential consumers to follow. Therefore, in the coupled network, the effect of open social media opinion leaders is less evident than signal-platform.

This study also has some practical implications. Companies should focus on increasing consumer trust in open social media platforms. The closed social media platform is based on offline social relationships, and friends’ connections and so on generally have a higher level of trust. Therefore, when travel information from multiple platforms affects consumers simultaneously, potential consumers are more likely to choose travel advertising information on a closed social media platform with higher trust over open social media with lower trust. Therefore, open social platforms should focus on improving social platform trust relationships, thereby increasing the cross-platform dissemination effect of open platforms. Simultaneously, for a better cross-platform publicity effect on the opening platform, the number of opinion leaders in travel advertising can be appropriately increased. However, how to determine the number of opinion leaders deserves further exploration.

At the same time, this paper also has some limitations. For example, this paper focuses on the difference in propagation properties between the opinion leader and the follower. In the future research, the properties of the opinion leader and the follower can be further considered, such as hobbies, social status, etc (Anagnostopoulos et al., 2022), which can be closer to the information propagation process in the real environment. In addition, this paper only considers the cross-platform dissemination process of competing advertising information in two online platforms. In the future, we can consider building a cross-platform competitive dissemination model of advertising in online-offline multi-layer networks (Ju et al., 2022), so as to better describe the actual dissemination process of competing advertising information.

## Data availability statement

The raw data supporting the conclusions of this article will be made available by the authors, without undue reservation.

## Author contributions

JC: conceptualization, formal analysis, and writing—original draft. HW: writing—review and editing. XC: conceptualization, methodology, and writing—review and editing. All authors contributed to the article and approved the submitted version.

## Funding

This work was supported in part by grants from the National Social Science Foundation of China(#22BGL236), National Natural Science Foundation of China (#72274132), and the Fundamental Research Funds for the Central Universities (JBK2201026).

## Conflict of interest

The authors declare that the research was conducted in the absence of any commercial or financial relationships that could be construed as a potential conflict of interest.

## Publisher’s note

All claims expressed in this article are solely those of the authors and do not necessarily represent those of their affiliated organizations, or those of the publisher, the editors and the reviewers. Any product that may be evaluated in this article, or claim that may be made by its manufacturer, is not guaranteed or endorsed by the publisher.
